# Object geometry serves humans’ intuitive physics of stability

**DOI:** 10.1038/s41598-024-51677-5

**Published:** 2024-01-19

**Authors:** Yaxin Liu, Vladislav Ayzenberg, Stella F. Lourenco

**Affiliations:** 1 Emory University, 36 Eagle Row, Atlanta, GA 30322 USA; 2 University of Pennsylvania, Philadelphia, PA USA

**Keywords:** Human behaviour, Perception

## Abstract

How do humans judge physical stability? A prevalent account emphasizes the mental simulation of physical events implemented by an intuitive physics engine in the mind. Here we test the extent to which the perceptual features of object geometry are sufficient for supporting judgments of falling direction. In all experiments, adults and children judged the falling direction of a tilted object and, across experiments, objects differed in the geometric features (i.e., geometric centroid, object height, base size and/or aspect ratio) relevant to the judgment. Participants’ performance was compared to computational models trained on geometric features, as well as a deep convolutional neural network (ResNet-50), none of which incorporated mental simulation. Adult and child participants’ performance was well fit by models of object geometry, particularly the geometric centroid. ResNet-50 also provided a good account of human performance. Altogether, our findings suggest that object geometry may be sufficient for judging the falling direction of tilted objects, independent of mental simulation.

## Introduction

Humans are exceptional at understanding the physical environment. Indeed, a hallmark of our intuitions about the physical world—our so-called ‘intuitive physics’—is the efficiency with which we estimate dynamic state changes of objects or events^1,2^. Human adults quickly, and accurately, determine where to place a coffee cup on a cluttered table or how to stack a pile of books. And even children as young as 3 months of age display a broad awareness of physical events, including the (implicit) understanding that an unsupported object should fall to the ground^3,4^. Despite our ease in reasoning about the physical environment, and a seemingly early sensitivity to object stability, much remains unknown about the mechanisms that shape the development of our intuitive physics judgments.

A long-standing theory, coming largely from developmental science, argues that our intuitions about the physical environment are based on acquired rules (or heuristics). From this perspective, our predictions about future events follow from our prior experiences, and these experiences form the basis for a system of rules that we apply to similar scenarios^5,6^. For example, if a half-supported cup falls from a kitchen table, then a half-supported plate on the table is also likely to fall. In this view, the similarity to a prior experience is determined via the perceptual features of objects and the larger scenes^7^. A learned rule is generalized to similar scenarios unless it becomes insufficiently diagnostic in the novel context^2,8^, at which point, learning continues by detecting violations of the learned rule and acquiring new rules relevant to the novel context.

More recently, an alternative theory based on mental simulation has become the predominant account of intuitive physics. Rather than the implementation of a specific rule, or rules, to a given scenario, this theory posits a dedicated ‘game engine’ in the mind, known as the intuitive physics engine (IPE)^9–11^. From the perspective of an IPE, humans are equipped with the parameters necessary to perform mental simulations in real time across a variety of (novel) scenarios^11^. Rules are not implemented but, instead, decisions about physical interactions are determined by probabilistic simulations of likely outcomes.

Despite the aforementioned differences, both accounts acknowledge the importance of perceptual inputs for intuitive physics judgments. It is believed that such inputs allow for assessing similarities across scenarios or constraining the mental simulation. In neither case, however, would such information adequately support physical reasoning. And yet, recent data using deep convolutional neural networks (DNNs) would seem to suggest otherwise^12–14^. For example, Conwell et al. showed that the visual representations learned by DNNs from image datasets were predictive of human performance on a block tower task in which participants judged the stability of stacked objects. This finding provides an important proof of concept—judgments of physical stability were possible from the perceptual inputs alone. Nevertheless, it is unclear precisely which inputs are relevant, especially in relation to humans’ judgments.

Here we provide a strong test of the possibility that our intuitions about the stability of objects are rooted in the perceptual primitives that constitute objects, particularly the geometric features^15^. Our approach is motivated by research on humans’ sensitivity to object geometry, which is present from early in life^16–18^ and may be phylogenetically ancient^19^. Moreover, object geometry is used to make predictions about dynamic events, such as causality^20,21^, continuity^22^ and motion trajectories^23–25^. Nevertheless, two important questions remain unanswered. First, when judging the physical stability of an object, which geometric features do we rely on? And second, is an analysis of object geometry, in the absence of mental simulation, sufficient for judging stability?

**Figure 1:**
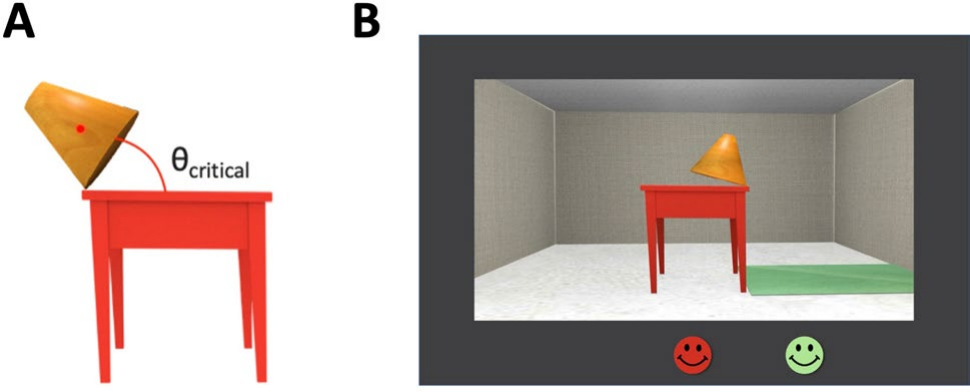
(**A**) Illustration of an object’s geometric centroid (represented by the red dot) supported by the edge of the table. When the object’s geometric centroid is vertically supported by the base (as in the current example), the object is equally likely to fall to the ground as it is to remain on the table. The angle between the object and its supporting base in this case is the critical angle. (**B**) An illustration of an experimental trial. Human participants were instructed to judge whether a tilted object would fall to the green mat or the red table. They responded by selecting the green or red face. (Faces were used to make the task more child-friendly for child participants).

One object property that may be particularly important for stability judgments is the geometric centroid. The geometric centroid, also known as an object’s center of mass, constitutes the unique point of an object on which the entire mass of the object is averaged. It can be considered a latent summary statistic, which is computed from a set of perceptible features (e.g., height, base, and aspect ratio). Under the influence of gravity, if the geometric centroid is supported by a base beneath it, then the entire object is supported (assuming uniform mass distribution), such that the entire weight of an object acts at its geometric centroid (see Fig. 1A).

Research with human participants has revealed that adult observers estimate the geometric centroids of 2D shapes and 3D objects with good precision^26,27^. It has even been shown that adults treat this information preferentially, attending to the geometric centroid during visual search, rather than local cues^28^, and treating the geometric centroid as a prototype of a spatial region^29^. Nevertheless, evidence for the role of the geometric centroid in stability judgments is lacking^26^. For example, Cholewiak et al.^26^ suggested that despite good accuracy in estimating the centroids of different objects (centroid estimation task), participants did not use the centroid to judge the critical angle of falling for a tilted object (stability task). This claim, however, was based on non-significant correlations between centroid estimation and stability tasks for a small sample of participants. Particularly challenging for this interpretation was that participants’ estimates across the two tasks scattered in the opposing direction. On the centroid task, participants underestimated the centroid, whereas on the stability task, their estimates of the critical angles of falling involved overestimation (i.e., “safe side” judgments). These differences in response bias may have reflected different task strategies, not the irrelevance of an object’s centroid for judging stability.

In the present study, we provide a two-fold approach to testing whether human observers—adults and children—judge object (in)stability according to the object’s geometric centroid. First, we tested adults and children on a psychophysical task that required judging the direction of falling for a tilted object. Participants were tested with carefully designed objects that allowed for examining the extent to which they relied on specific geometric features. Moreover, we compared the judgments of adult and child participants to shed light on potential developmental similarities and differences. Although previous research has found sensitivity to the geometric centroid among adults, it is unclear whether children have a similar tendency, including using this information to estimate falling direction. Second, we compared participants’ behavioral responses to two types of models. One type, which we refer to as the geometric models, was based on measurable geometric features (i.e., base size, centroid position, height, or top/bottom [T/B] ratio). The other type was ResNet-50, a DNN pretrained on ImageNet, which is known for its success in object classification^30^. Together, the human-model comparisons provide a strong test of whether, and what types of, perceptual features are sufficient for supporting judgments of falling direction.

Across two experiments, participants were presented with tilted objects and questioned whether the object would fall to the ground or onto the table (see Fig. 1B). More specifically, because the objects were tilted, participants’ decisions were specific to the direction of falling, not whether the objects might remain in place. We assessed the precision with which adults and children estimated falling direction across a range of conditions, including when specific geometric features (e.g., object height) were irrelevant to the judgment. Because geometric features are correlated with one another, it is not possible to isolate each feature (e.g., centroid). Thus, we varied geometric features iteratively across conditions so as to hone in on which geometric features were most relevant to participants’ judgments.

Model performance was tested using only the geometric features of objects (geometric models) or the visual properties of scenes (ResNet-50) and did not incorporate any rules or simulations regarding object falling. If geometric models well approximate human performance, then this would suggest that geometric properties specific to the target object are sufficient to account for human judgments of falling direction. If ResNet-50 also well approximates human performance, then this would additionally suggest that the perceptual inputs from a DNN may be sufficient for judgments about object falling without a simulation mechanism.

## Results

### Analytical approach

The intuitive physics of object (in)stability was assessed with a psychophysical task that required judging whether a tilted object would fall to the ground or onto the table. Participants were instructed that the object was unstable so their decision was to judge the side to which it would fall (i.e., green mat or red table). For human participants, each experimental trial generated a binary response (1 = “green mat”; 0 = “red table”). Classification responses were gathered for each angle, object, and individual participant. Psychometric functions (GLMs) predicting the proportion of falls to the green mat were then fitted to participants’ responses.

Object (in)stability judgments of geometric models and ResNet-50 were tested by iteratively training a support vector machine (SVM) classifier on 10 stimulus objects with a linear kernel, and then testing stability judgments for one left-out object (1000 stratified resampling). For the geometric models, the numeric values of each geometric feature and the tilted angle were inputted as features to the SVM. For ResNet-50, features were obtained from the penultimate layers (AvgPool) of a pretrained ResNet-50 architecture (see “Methods and materials”).

Psychometric functions fitted to the classification data of human adults, children, geometric models and ResNet-50 were modeled using the QuickPsy R package^31^. The point of subjective equality (PSE), which allows for assessing the threshold of the critical angle when the object is judged equally likely to fall to either side (0.50 proportion of falls to the green mat), was extracted from a bootstrapping of 1000 resamples. Mean PSEs of each object and individual participants/models were outputted for group comparisons in analyses of variance (ANOVAs). Hypotheses were additionally evaluated using bayes factors (BF), obtained by using the BayesFactor R package. BF_10_ is the bayes factor in favor of an alternative hypothesis against a null hypothesis, whereas BF_01_ is the bayes factor in favor of a null hypothesis against an alternative hypothesis. See Supplemental Information (SI) for accuracy (Supplemental Figs. 1 and 2) and psychometric fitting (Supplemental Figs. 3 and 4).

Preliminary analyses of children’s performance revealed no differences between 5- and 6-year-olds (see SI). Thus, all subsequent analyses on child participants combined the two age groups. Because adult participants completed Experiments 2A and 2B in counterbalanced order, analyses of order were performed. There was no significant order effect (see SI) and, thus, data were subsequently collapsed across this variable.

### How accurate are human observers and computational models at judging direction of falling?

We first compared PSE estimates of humans and models against the ground truth values of the objects’ critical angles (see SI for analytical calculations and scripts; Supplemental Table 1) using one-sample t-tests. The PSE estimates of humans and models were subtracted from ground truth values, such that the differences were tested against zero. Models were the geometric models: base, centroid, height, and top/bottom ratio (T/B ratio). Additionally, we included ResNet-50. See “Methods and materials”.

**Figure 2:**
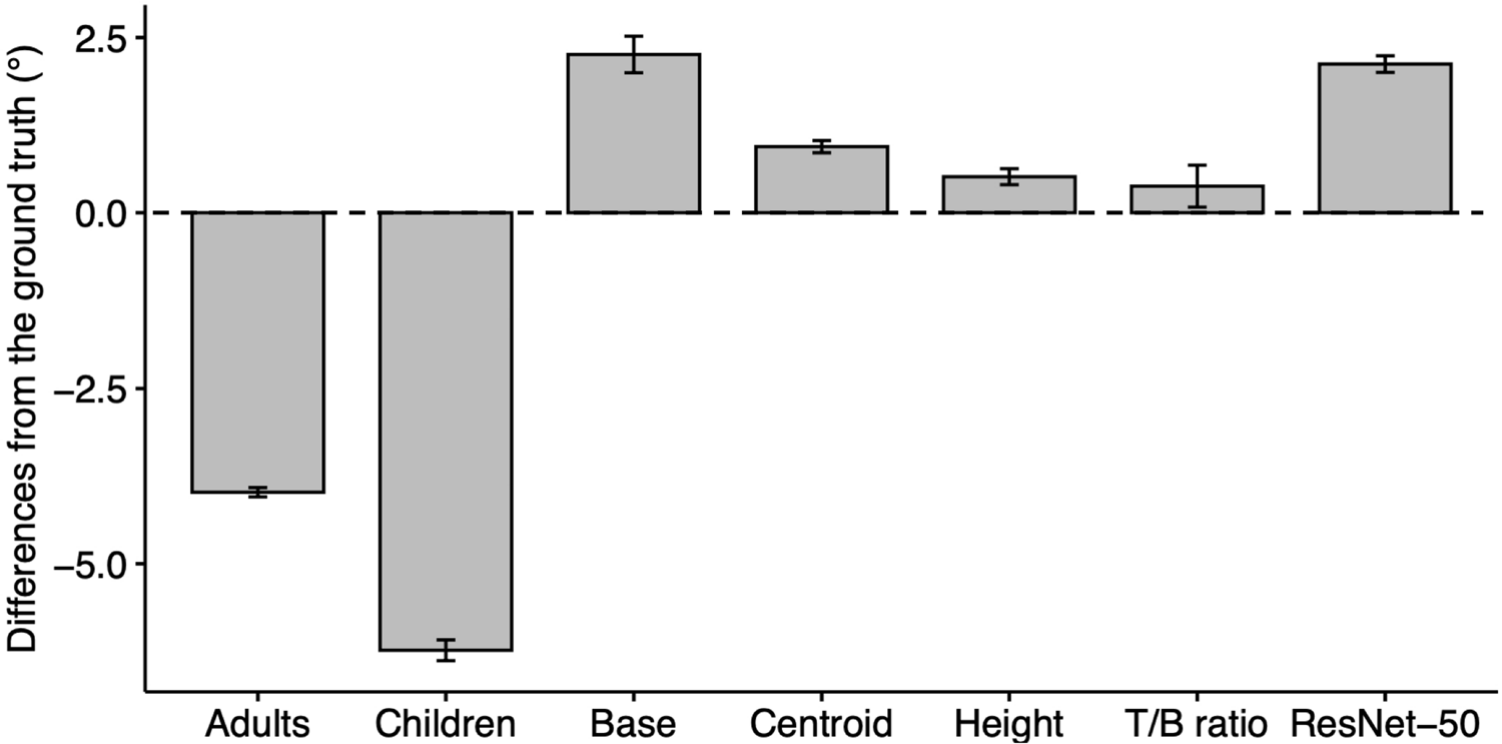
Mean differences between PSEs and the ground truth plotted for human participants (adults and children) and each of the models (base, centroid, height, T/B ratio, and ResNet-50). Error bars represent 95% bootstrapped CIs. These results are collapsed across experiments. See Supplemental Fig. 2 for plots by object and experiment.

Across Experiments 1 and 2, both adults and children showed significantly lower PSEs relative to the ground truth (*ps* < 0.001; Fig. 2), though adults displayed better accuracy than children (see SI for results). That human observers underestimated stability by predicting objects would fall to the green mat at smaller angles than the ground truth suggests a ‘safe-side bias’, as reported in other research ^32^. However, models did not display such a bias (Fig. 2). In fact, the PSEs of all models were closer to the ground truth than the PSEs of humans (all *ps* < 0.001). Additionally, all models showed significantly higher PSEs relative to the ground truth (*ps* < 0.001).

### Which geometric features do humans favor when judging falling direction?

#### Experiment 1A

**Figure 3:**
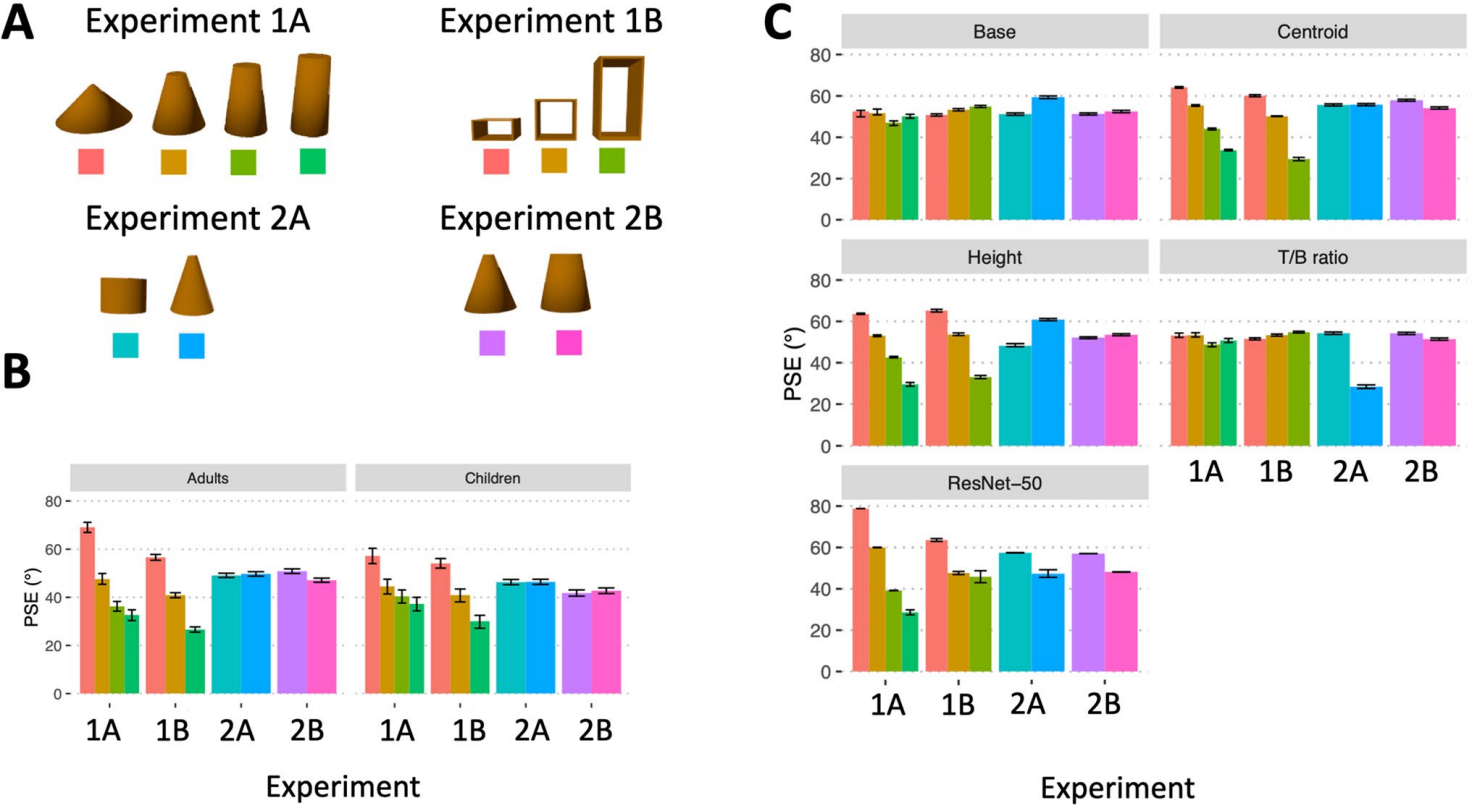
Stimuli and results for Experiments 1 and 2. (**A**) The eleven objects used across experiments. Color patches correspond to objects in the data panels (**B** and **C**). (**B**) Point of subjective equality (PSE) estimates of critical angles plotted for adult and child participants in each experiment. (**C**) PSE estimates plotted for each of the geometric models and ResNet-50 in each experiment. Error bars represent 95% bootstrapped CIs. Adults and children performed comparably across all experiments, except in Experiment 2B in which children did not show differences in PSE estimates between the two objects (whereas adults did). Among the geometric models, the centroid model showed performance that was qualitatively similar to human participants across all conditions. All other geometric models differed from humans in at least one of the conditions by displaying opposite response profiles. ResNet-50 showed qualitatively similar response profiles to humans, except in Experiment 2A.

Objects in Experiment 1A were designed to covary in their geometric properties, so that differences in the objects’ centroids were maximized (see Fig. 3A). If human observers are sensitive to the geometric centroid of objects, and use them to judge falling direction, then their PSE estimates should scale according to the objects’ critical angles. To test this prediction, we performed linear trend analyses as regressions for child and adult participants. Analyses revealed significant linear trends for both adults (*b* = − 31.40, *p* < 0.001, ${\eta }_{p }^{2}$ = 0.83) and children (*b* = − 17.19, *p* < 0.001, ${\eta }_{p }^{2}$ = 0.22), as predicted if their judgments depended on the geometric centroids of the objects (Fig. 3B). However, because the objects also varied in other geometric properties, such as base size and height, it remains possible that participants instead used other object features when judging falling direction.

#### Experiment 1B

In Experiment 1B, objects were identical in base size and T/B ratio, leaving only the objects’ heights and centroids to vary (see Fig. 3A). If participants rely on object height or the centroid, then their PSE estimates should continue to scale according to the critical angle, as in Experiment 1A. If, instead, they rely on base size and/or T/B ratio, they should treat the objects similarly. In addition, the objects here were hollow, allowing for an important test of generalization. As in Experiment 1A, linear trend analyses of participants’ PSE estimates revealed significant linear trends for both adults (*b* = − 31.01, *p* < 0.001, ${\eta }_{p }^{2}$ = 0.81; Fig. 3B) and children (*b* = − 19.99, *p* < 0.001, ${\eta }_{p }^{2}$ = 0.29; Fig. 3B). Taken together, these findings provide specific support for the use of object height or the geometric centroid, even when the objects are non-solid.

In Experiment 2, we created solid objects in which the two objects had the same (Experiment 2A) or different (Experiment 2B) critical angles (see Fig. 3A). More specifically, in Experiment 2A, the objects had the same geometric centroid (different object height), and in Experiment 2B, the objects differed in geometric centroid (same object height). By varying the objects in this way, we tested the extent to which the geometric centroid (independent of height alone) is used for judging object stability. We predicted that if the geometric centroid is relevant when judging falling direction, then objects with comparable centroids (Experiment 2A) would not differ in their critical angles. However, objects with different geometric centroids (Experiment 2B) should be treated differently, despite being identical in height. In both cases, base size was identical for the two objects and, thus, uninformative for these judgments.

#### Experiment 2A

We found that the PSE estimates of adults (*M*_*difference*_ = − 0.9°, *t*[36] = − 1.04, *p*_*corrected*_ = 0.611, *d* = 0.35, 95% CI [− 2.83, 1.09], BF_10_ = 0.37) and children (*M*_*difference*_ = 0.2°, *t*[36] = − 0.23, *p*_*corrected*_ = 0.999, *d* = 0.06, 95% CI [− 2.05, 1.68], BF_10_ = 0.23) did not statistically differ for the two objects (Fig. 3B). Furthermore, a mixed-factor ANOVA, with object as the within-subject factor and age group as the between-subject factor, revealed no significant main effects (object: *F*_[1, 36]_ = 0.83, *p* = 0.367, ${\eta }_{p }^{2}$ = 0.023, BF_10_ = 0.36; age group: *F*_[1, 36]_ = 3.08, *p* = 0.088, ${\eta }_{p }^{2}$ = 0.079, BF_10_ = 1.2) or interaction between factors (*F*_[1, 36]_ = 0.35, *p* = 0.556, ${\eta }_{p }^{2}$ = 0.010, BF_10_ = 0.38). These findings suggest that neither children, nor adults, differentiated the objects’ critical angles, as predicted if the geometric centroid is necessary for judging falling direction. These findings also suggest that height and T/B ratio are subordinate to the geometric centroid, at least with these objects, because participants did not differentiate between them. If observers had relied on object height or T/B ratio, which differed between the two objects, then they would have treated the objects differently with respect to their critical angles. They did not.

#### Experiment 2B

Unlike Experiment 2A, the two objects in Experiment 2B differed in their geometric centroids, resulting in a small change in their critical angles (difference = 8.5°; see Fig. 3A). We predicted that if human observers rely on an object’s centroid to judge falling direction, and the centroids between objects are sufficiently discriminable, then observers should accurately estimate the angle of tilt at which objects fall to the ground.

A mixed-factor ANOVA, with object type as the within-subject factor and age group as the between-subject factor, revealed significant main effects of object (*F*_[1, 39]_ = 4.58, *p* = 0.039, ${\eta }_{p }^{2}$ = 0.11, BF_10_ = 1.26) and age group (*F*_[1, 39]_ = 17.15, *p* < 0.001, ${\eta }_{p }^{2}$ = 0.31, BF_10_ = 101.87), as well as a significant interaction between these two factors (*F*_[1, 39]_ = 15.65, *p* < 0.001, *η*_*p*_^*2*^ = 0.29; BF_10_ = 1.26). Post hoc analyses of the interaction (Bonferroni corrected) revealed that adults’ PSE estimates for the two objects were significantly different (*M*_*difference*_ = 3.80°; *t*[39] = 4.07, *p*_*corrected*_ < 0.001, *d* = 1.3, 95% CI [1.62, 5.98], BF_10_ = 35.61; Fig. 3B), and together with the results of Experiment 2A (where T/B ratio differed between objects but did not influence performance), these findings suggest that adult observers favor the geometric centroid when reasoning about tilted objects.

Yet children’s PSE estimates for the two objects were not significantly different (*M*_*difference*_ = − 1.13°, *t*[39] = − 1.37, *p*_*corrected*_ = 0.357, *d* = 0.44, 95% CI [− 3.06, 0.80], BF_10_ = 0.52; see Fig. 3). How should this result be interpreted? Although children differed from adults in this experiment, they performed comparably to them in Experiment 1, which suggests two possibilities for their performance here. The first possibility is that children also rely on object centroids when judging falling direction, but their representations are less precise than those of adults. The second possibility is that children instead rely on other geometric features. Given that children did not distinguish objects in Experiment 2A, where other features were available, this possibility seems unlikely. Subsequent comparisons between human and model performance will shed further light on these possibilities.

### Models’ estimates of falling direction

#### Experiment 1

Analyses of models’ performance in Experiment 1 confirmed that a subset of geometric models and ResNet-50 judged falling direction according to the critical angle of objects. Trend analyses (with polynomial contrasts) were performed as regressions on each of the geometric models (base, centroid, height, and T/B ratio) and ResNet-50. In Experiment 1A, these analyses revealed that all models, except the base model (*b* = 0.19, *p* = 0.092), exhibited PSE estimates that decreased as a function of object type (centroid [*b* = − 24.21, *p* < 0.001]; height [*b* = − 25.31, *p* < 0.001], T/B ratio [*b* = − 4.22, *p* < 0.001]; ResNet-50 [*b* = − 38.33, *p* < 0.001]); see Fig. 3C. These patterns were qualitatively similar to those of human participants. In Experiment 1B, centroid [*b* = − 23.86, *p* < 0.001], height [*b* = − 25. 72, *p* < 0.001], and ResNet-50 [*b* = − 12.42, *p* < 0.001]) models similarly exhibited PSE estimates that decreased as a function of object type, like humans. Base (*b* = 2.97, *p* < 0.001) and T/B ratio (*b* = 2.18, *p* < 0.001) models, however, showed the opposite trends (Fig. 3C), suggesting that base and T/B ratio were not correctly used to judge the direction of falling.

#### Experiment 2

Analyses of model estimates in Experiment 2A, where the critical angle of falling to the ground was equated across the two objects, revealed that the centroid model did not differentiate between the PSE estimates of the two objects (Fig. 3C). Specifically, like humans, the PSEs for the two objects were not significantly different for the centroid (*M*_*difference*_ = − 0.15°) model. Unlike humans, however, the models of base (*M*_*difference*_ = − 8.20°), height (*M*_*difference*_ = − 12.63°), and T/B ratio (*M*_*difference*_ = 25.85°) incorrectly discriminated between the two objects’ PSE estimates (Fig. 3C). Likewise, ResNet-50 (*M*_*difference*_ = 8.40°) showed a significant difference in PSE estimates (Fig. 3C).

Analyses of model estimates in Experiment 2B, where the objects differed in the geometric centroids, albeit minimally, revealed that the centroid model (*M*_*difference*_ = 3.84°), T/B ratio model (*M*_*difference*_ = 2.75°), and ResNet-50 (*M*_*difference*_ = 8.8°) correctly showed differences in PSE estimates between the two objects, such that a more stable object had a larger PSE estimate than a less stable object. By contrast, base (*M*_*difference*_ = − 1.18°) and height (*M*_*difference*_ = − 1.40°) models showed opposite patterns in PSE estimates (Fig. 3C).

Taken together, the findings of Experiment 2 demonstrate reliable performance by the centroid model, such that it treated the two objects as equivalent when the critical angle was indistinguishable (Exp. 2A) but as different when they differed, even minimally (Exp. 2B). The other geometric models classified objects according to the trained geometric feature when this information was available, but this performance was generally inaccurate with respect to falling direction (except for T/B ratio in Exp. 2B). The performance of ResNet-50 demonstrates that inputs from raw images are sufficient for distinguishing between two objects with respect to falling direction, though performance was less accurate in Experiment 2A than Experiment 2B.

### Using the geometric models and ResNet-50 to inform humans’ performance

Previous research has raised doubts about human observers’ reliance on the geometric centroid for predicting object stability^26^. However, the aforementioned findings suggest that the geometric centroid is indeed used for judging the direction of falling. Next, we provide further support for this claim by comparing human observers to computational models trained to judge falling direction on the basis of different geometric features or raw inputs of images.

To test whether human performance would be best matched by the centroid model or the models of base, height, and T/B ratio, we examined whether human participants and geometric models exhibited similar response patterns across trials (for analyses of individual models, see SI). To this end, we performed multiple regression analyses with human performance as a dependent variable and the geometric models’ performance as independent variables. Both human and model performance were indexed by the proportion of falling responses (the number of responses to the green mat/the number of trials) of each object at each tilt. In addition, we obtained Bayes factors to compare the fit of the full model (containing all predictors) against models without the predictor of interest.

#### Geometric models

Regression analyses revealed that human performance was well fit by all the geometric models altogether (base, centroid, height and T/B ratio; Adults: *R*^*2*^ = 0.88; Children: *R*^*2*^ = 0.83). Moreover, these analyses revealed that the centroid explained the greatest amount of unique variance in adults and children; the regression coefficients were significantly different from zero (adults: *b* = 0.52, *p* < 0.001; children: *b* = 0.24, *p* = 0.004). The other geometric feature that explained a significant amount of variance in both age groups was height (adults: *b* = 0.28, *p* = 0.002; children: *b* l = 0.22, *p* = 0.013). Base size was not a significant predictor of either adults’ (*b* = − 0.02, *p* = 0.787, BF_01_ = 16.7) or children’s (*b* = 0.12, *p* = 0.122, BF_01_ = 3.97) responses. Similarly, the T/B ratio was also not a significant predictor of either adults’ (*b* = 0.07, *p* = 0.327, BF_01_ = 9.90) or children’s (*b* = 0.10, *p* = 0.134, BF_01_ = 4.26) responses. These findings are consistent with the aforementioned behavioral responses in which adults relied on the geometric centroid for judging falling direction and they suggest that children engage in a similar strategy. These findings also suggest that participants’ may have relied on object height as well, though this effect may reflect the correlation between the geometric centroid and object height in a subset of objects (as in Experiment 1).

**Figure 4:**
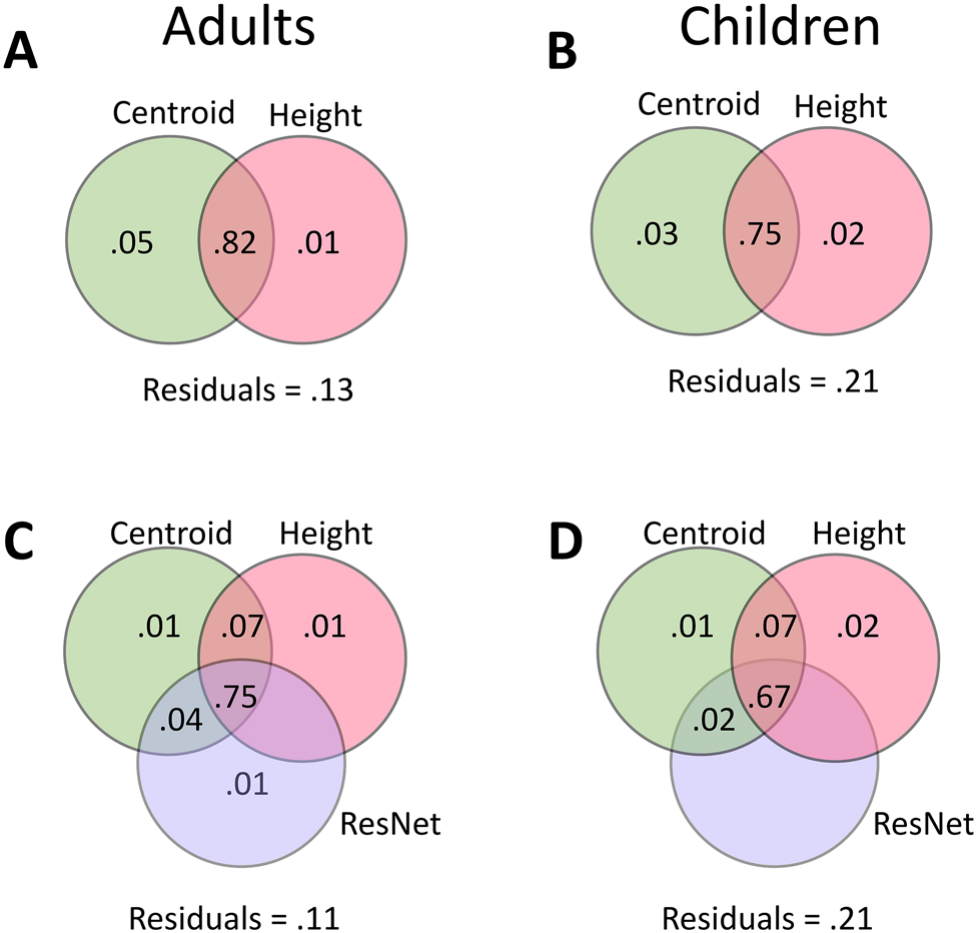
(**A** and **B**) Variance partitioning of geometric models for adults and children, excluding the base and T/B ratio models (neither of which significantly predicted human performance). (**C** and **D**) Variance partitioning of ResNet-50 and geometric models for adults and children (again, excluding base and T/B ratio models). Numerical values in overlapping and non-overlapping regions of the Venn diagrams indicate the proportion of shared and unique variance, respectively, in the corresponding analyses. Values smaller than 0.01 are not displayed.

In general, the geometric centroid co-varies with object height. Given this algorithmic overlap, we tested the prediction of shared variance among the geometric models using variance partitioning analyses (VPAs^33^). In these analyses, we used centroid and height to explain human performance (see Fig. 4A,B). In adults, the shared variance for the two predictors explained most of the total explainable variance (93.60%). In children, the shared variance for the two predictors was also the majority of the total explainable variance (94.37%). Additionally, through a permutation test with 1000 permutations, we found that the unique variance explained by the centroid was significantly different from that of height among adults (*M*_difference_ = 3.5%, *p* < 0.001), but not among children (*M*_difference_ = 1.0%, *p* = 0.159). These findings were generally consistent with our regression analyses above, but they also demonstrate the privileged role of the geometric centroid in accounting for stability judgments, particularly among adults.

Taken together, these findings not only demonstrate good fit between the geometric models and humans’ judgments at different stages of development, but they also demonstrate shared variance between centroid and height. In addition, these findings confirm that the objects’ centroids are predominant in adults’ judgments of object (in)stability, and they point to the additional influence of object height in children.

#### ResNet-50

Recent research suggests that DNNs, such as ResNet-50, pre-trained on the ImageNet dataset, can judge stability, even of multi-object towers, with comparable performance to that of adults^13,34^. These results have been used to argue against a mental simulation account such as the IPE^9^. Here we further test this possibility with stimuli designed to isolate the role of individual geometric features in both adult and child samples.

Regression analyses comparing the response patterns of humans and ResNet-50 revealed relatively good fit to human performance in both age groups (adults: *R*^*2*^ = 0.80, *b* = 0.79, *p* < 0.001; children: *R*^*2*^ = 0.69, *b* = 0.60, *p* < 0.001). Moreover, when including ResNet-50 and geometric models in VPA to examine the extent of shared variation, we found that the shared variation between models explained the majority of the total explainable variance in both adults (83.13%) (see Fig. 4C) and children (83.21%) (see Fig. 4D). However, there were no significant differences among the unique variance explained by centroid, height, and ResNet-50 models using a permutation test (*ps* > 0.05). The shared variance between geometric models and ResNet-50 would seem to suggest that the success of ResNet-50 in predicting falling direction may reflect the extraction of geometric features such as the centroid and height of the object.

### Correlations between ResNet-50 and the geometric models

**Figure 5:**
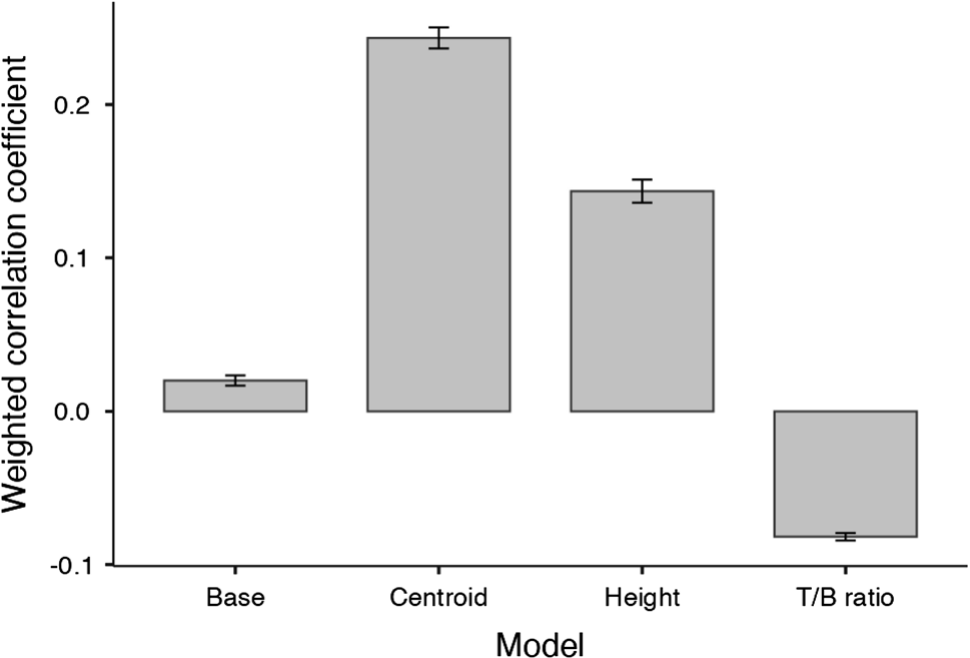
Weighted correlation coefficients between each of the geometric models and ResNet-50 feature output (15 principal components). Error bars represent 95% CIs.

To better understand the nature of the shared variance between models, we next examined the relation between the raw outputs of ResNet-50 and the geometric models, independent of task performance. The raw outputs of ResNet-50 were extracted from the penultimate layer, and the features of geometric models were the feature datasets (see Methods). Because the raw outputs of ResNet-50 were high dimensional data, a principal components analysis (PCA) was employed to reduce the dimensionality of the feature output from the pre-trained ResNet-50 while retaining 90% of the variance, yielding the 15 largest principal components. This is consistent with the possibility that the inputs relevant to stability judgments may be encoded by a subset of dimensions (15 dimensions) in the DNN^35^. These components were then selected and subsequently tested for correlations between these components and the geometric models in the feature dataset. Correlation coefficients were bootstrapped from 1000 resamples for the purpose of deriving confidence intervals in subsequent analyses. Each corresponding coefficient was weighted by the variance explained by each principal component from 1 to 15. The final weighted coefficients were mean correlations from 15 components across 1000 resamples. Analyses of one-sample t-tests revealed that the weighted correlation coefficient of each model (base, centroid, height, and T/B ratio) was significantly different from 0 (*ps* < 0.001; see Fig. 5).

An ANOVA was also performed to test for differences between the coefficients of the models. This analysis revealed a significant main effect of model type (*F*[3, 3996] = 364.80, *p* < 0.001, ${\eta }_{p }^{2}$ = 0.83). Post hoc analyses showed that the centroid model had a higher correlation coefficient than all other models (*ps* < 0.001). Altogether, these findings suggest sensitivity to object geometry within ResNet-50, particularly the geometric centroid.

## Discussion

A fundamental question about humans’ intuitive physics concerns the computations used to reason about the status of a falling event. By varying object geometry and comparing the performance of human adults and children to different computational models, we found that the use of object-centered geometry allowed for intuiting how an unstable object would fall. The predominant theory of intuitive physics appeals to an intuitive physics engine (IPE) in which probabilistic mental simulation is necessary for judging object stability. The present findings, however, argue against the necessity of mental simulation, at least when judgments are constrained to simple physical events. Moreover, our data suggest that the perceptual inputs related to object-centered geometry, particularly the object’s geometric centroid, are sufficient for predicting a falling event.

The present data suggest a privileged role for the geometric centroid in stability judgments. Indeed, it builds upon other research that demonstrates that at least adults are highly sensitive to the centroids of objects^27,36^. Here we show that this sensitivity is present in young children as well, though there may be developmental change in the precision of these estimates. Previous research has shown the estimation of centroids may be established through haptic experiences (i.e., grasping or lifting)^37,38^. Given the relation to the center of gravity, a sensitivity to the geometric centroid may afford efficient interactions with physical objects. Yet an important question is how the centroid is computed by the visual system. Accumulating evidence suggests that the computations are based largely on object shape^39,40^. Psychophysical data point to the relevance of a symmetry axis, or an internal shape skeleton, for localizing the centroid, particularly in symmetrical objects^16,41^. From this perspective, the human visual system extracts a low-dimensional shape representation based on one or more internal axes, on which the centroid overlaps^39^. Thus, the centroid comprises a point within the global shape rather than the local features of the object.

Is it possible that ResNet-50 also computes the geometric centroid? The finding that the feature outputs of ResNet-50 were most strongly correlated with the centroid model points to the intriguing possibility that the geometric centroid within this neural network model may reflect a property of the raw visual inputs. But what would constitute the relevant inputs? Unlike human perception, global shape information is not readily represented by DNNs^42,43^. Thus, it would seem more plausible that the relevant inputs comprise the local geometric cues (e.g., height and base size), which are correlated with objects’ centroids. If so, this would suggest an important difference between ResNet-50 and human observers. ResNet-50 may not classify falling direction in the exact same way as humans. For example, in Experiment 2A of the present study, one would want to filter out height information to accurately judge the falling direction, which even young children seemed capable of doing. By contrast, we speculate that ResNet-50 may have been overly reliant on this information, which would result in prediction errors.

Recently, Bass et al.^44^ proposed that human observers rely on ‘partial’ simulations, in which only a subset of events is predicted forward in time. For example, with a tower of blocks, this type of mental simulation would not act upon the entire motion trajectory; rather, only the parts that are relevant to the falling event would be simulated. Might the participants in the present study have similarly relied on partial, rather than full, simulation? Unfortunately, our data do not provide a definitive answer to this question. On the one hand, participants’ RTs did not differ between long and short distances (see Supplemental Fig. 4A,B), as would be predicted by an IPE in which stability judgments should be captured by the simulation time needed for the judgments. On the other hand, participants’ RTs did increase with angular distance (see Supplemental Fig. 5A,B), though such effects could instead reflect difficulty across trials^45^. It is also possible that our task induced some degree of implied motion^46,47^, which could account for the relation between RT and angular distance, rather than partial simulation per se.

There are well-known conditions in which mental simulation is used by human observers to reason about the physical relations of objects. For example, when deciding which direction a gear in a row of gears will move, many observers report rotating the gears in opposite directions in their minds^48^. Reaction times that track with the number of gears in the problem corroborate this introspection (as do participants’ gestures that mimic the gears’ movements during problem solving). Although it is unclear whether the mental simulation in this type of problem is implemented by a probabilistic IPE, there may be conditions in which full, or partial, probabilistic simulations are necessary for judging object stability or other physical events. The present task only involved a single object, so it is possible that judgments about the relations between two or more objects would benefit from probabilistic simulations^9,49, 50^. It is also possible that physical events involving objects with unexpected mass properties would be judged more accurately by way of an IPE. More research will be needed to compare these different conditions to determine the extent to which perceptual inputs related to object geometry are sufficient for judging stability specifically and physical events more generally.

What about rule learning? Although proponents of an IPE argue against rule learning as an account of humans’ intuitive physics, the present findings are not inconsistent with rule learning. That is, participants’ reliance on the geometric centroid could constitute a type of rule on which to base judgments of object stability. And, indeed, research with human infants would seem to support learning of relevant geometric features. In the case of object stability, it has been argued that children first acquire a rule about the amount of contact between an object and its supporting base and then transition to one about the proportion of contact^51^. Although more direct evidence is needed to determine whether infants, like older children and adults, rely on the geometric centroid for judging stability, it is possible that experience with objects under different conditions of stability allows observers to learn the rules associated with object features for making falling predictions. Alternatively, the primacy of particular geometric features might serve to constrain learning, such that attention directed to the geometry of objects may lead to better predictions of object stability early in the learning process and promoting subsequent learning in novel scenarios. Evidence from infant studies suggests that infants discover relevant features that they map to particular outcomes. Such feature discovery is coupled with statistical learning that serves regularity detection^52,53^. Consistent with this approach is recent research using a deep learning model of intuitive physics in which it was found that a ‘module’ trained on perceptual (visual) inputs of physical scenes constrained predictions about possible versus impossible events^15^.

Although humans’ intuitions about the physical world have long been lauded, much debate remains about how such intuitions are achieved. The present findings provide a novel demonstration of the role of object-centered geometric features in the intuitive physics of human adults and children. Altogether, our work suggests that adults and children predict the falling direction of tilted objects using geometric properties, particularly the object’s geometric centroid. Moreover, the extent to which participants’ responses were well modeled by both the geometric models and ResNet-50 suggests that mental simulation may be unnecessary, at least on a task constrained to judgments of falling direction. Future research would do well to establish the boundary conditions upon which object-centered representations are sufficient for predicting physical events and when simulation may be needed.

## Materials and methods

### Participants

Thirty-seven children and 40 adults participated in Experiment 1: 22 children (12 girls, *M*_*age*_ = 6.04 years, range = 5.18–7.12 years) and 20 adults (15 women, *M*_*age*_ = 18.4 years) in Experiment 1A; 15 children (11 girls, *M*_*age*_ = 6.24 years, range = 5.07–6.93 years) and 20 adults (13 women, *M*_*age*_ = 18.6 years) in Experiment 1B.

Forty-eight children participated in Experiment 2: 23 in Experiment 2A (11 girls, *M*_*age*_ = 5.90 years; range = 5.16–6.76 years) and 25 in Experiment 2B (13 girls, *M*_*age*_ = 6.02 years, range = 5.16–7.00 years). Eighteen adults completed both Experiments 2A and 2B (15 women, *M*_*age*_ = 19.1 years) in counterbalanced order.

Sample sizes were determined a priori using a power analysis via the mixed ANOVA function of the BUCSS R package^54^. Effect size (*N* = 13, ${\eta }^{2}= 0.81$) was based on previous findings^26^. The primary effects of interest were a within-subject effect of object type, a between-subject effect of age group, and an interaction between object type and age group (alpha level = 0.05, power = 0.8, assurance level = 0.8). We recruited a larger number of participants than the minimum sample size to ensure that a sufficient number remained in the event of attrition.

The final sample excluded child participants who failed to follow instructions. Data unusable for model fitting were excluded from the analyses (see SI for individual plots). The final sample consisted of 76 child participants (Experiment 1A: *N* = 19; Experiment 1B: *N* = 14; Experiment 2A: *N* = 20; Experiment 2B: *N* = 23). No adult participants were excluded. Written informed consent on behalf of child participants was obtained from their legal guardians. Adult participants provided written informed consent for themselves. Child participants received a small gift for their participation. Adult participants received course credit. The Institutional Review Board (IRB) at Emory University approved all procedures, and all methods and procedures were performed in accordance with the regulations established by the IRB.

### Stimuli

Stimuli were generated using Blender 3D software (v.2.7). All objects were rendered with a smooth texture for a uniform surface. Object dimensions were in blender units. See OSF^55^ for stimuli within a feature dataset. The task was programmed in PsychoPy2^56^ and presented on an HP touchscreen desktop computer (resolution: 1920 × 1080 px) for children and on a Dell desktop computer (resolution: 1280 × 1024 px) for adults.

#### Experiment 1A

Stimuli consisted of four 3D objects. Objects differed in height, base size, and top/bottom (T/B) ratio, resulting in four distinct geometric centroids and critical angles. Volume was equivalent across objects.

#### Experiment 1B

Stimuli consisted of three 3D objects with hollow interiors. Objects differed in height, resulting in three distinct geometric centroids and critical angles. Base size and T/B ratio were equivalent across objects.

#### Experiment 2

In Experiment 2A, there were two 3D objects. The objects differed in height and T/B ratio, but not base size. The objects were equivalent in geometric centroids and critical angle. In Experiment 2B, there were two 3D objects. The objects differed in T/B ratio, but were identical in height and base size, resulting in different centroids and critical angles (difference = 8.5°).

One concern is that children in Experiment 2A may not have discriminated the objects on the basis of height. To rule out this possibility, children were given ‘height check’ trials where they judged which of the two objects (same tilted angle) was taller (sequential presentation; counterbalanced order; 2 trials total). Analyses of these trials confirmed that children discriminated the two objects according to their height, *t*(21) = 3.17, *p* = 0.005, *d* = 0.68.

### Task procedure

Human participants were instructed to judge whether a tilted object would stay on the table or fall to the ground. Both child and adult participants first completed four practice trials in which an object placed on the left or right side of the tabletop was tilted at an angle of 15° or 110° from the table’s edge (side and angle counterbalanced across trials; randomized order). In the child version of the task, children were first shown a real 3D object on the edge of a table to ensure that they subsequently understood the scenario onscreen. On each trial, child participants were asked to touch the corresponding smiley face onscreen to respond. A red smiley face indicated that the object would fall towards the red table and a green smiley face indicated that the object would fall onto the green mat below. Corrective feedback was provided during the practice trials in the form of a video showing the corresponding event (e.g., the object falling to the floor). The test trials that followed were identical to the practice trials, except that no corrective feedback was provided. The task included four evenly spaced breaks. The adult version of the task was identical, except that adult participants used a computer mouse to respond. Responses and RTs were recorded.

For each test trial, an object was presented at a tilted angle. The range of the tilted angle varied (Experiment 1A [176 trials]: 5°–105°, in increments of 10°; Experiment 1B [156 trials]: 20°–80°, increments of 5°]; Experiment 2 (A & B) [156 trials each], varied ranges, in increments of 5°). The presentation side of the object (left and right) was counterbalanced.

Independent groups of child and adult participants completed each experiment except for Experiment 2, where adult participants completed both Experiments 2A and 2B (counterbalanced order). For child participants, we included an additional height check at the end of Experiment 2A to ensure that children were able to discriminate between the two objects. In the height check, the objects, with the same tilted angle, were presented sequentially (counterbalanced order). Participants were asked to judge which object was taller.

### Model descriptions

We trained a linear support vector machine (SVM) classifier, a type of supervised learning, where the classifier was trained to discriminate fall responses in each scene. We tested four geometric features of objects—object height, base size, T/B ratio, and the geometric centroid—using a feature dataset. We also included a DNN to extract features from images using scene image datasets.

#### Scene image set

The scene dataset consisted of 138 stimulus images from the psychophysical experiments, including a total of 11 objects and each angle increment from one side only. The image sets were used in a pre-trained ResNet-50 for feature extraction. Images were transformed to RGB channels, gray scaled and resized to 224 by 224 px. Images were cropped so that only an object and the edge of a table was visible from a given scene.

#### Feature dataset

The feature dataset consisted of numerical data of each of the features (height, base, T/B ratio, and geometric centroid) from a total of 11 objects and each angle increment across the two experiments (4 conditions). All numerical data used standard Blender units.

##### Base

The base was the width of an object’s bottom horizontal extent.

##### Height

The height was an object’s vertical extent.

##### Centroid

The centroid of an object was analytically computed (see SI Table 1)^55^ and analyzed as the vertical distance between the base and the position of the centroid.

##### Top/bottom (T/B) ratio

The T/B ratio was the ratio between an object’s top and bottom radii.

### Model analyses

All model analyses were performed using the Scikit-learn and PyTorch libraries (Python 3.6).

#### ResNet-50

We used a pre-trained deep convolutional neural network (DNN), ResNet-50, which is trained on millions of images from the ImageNet database^57^ and is successful in classifying object categories^30^. The last layer of ResNet-50 was a fully connected layer producing 1000 class labels. The first convolution layer takes an input image of 224 × 224, which is the dimension of the image sets. Features were extracted from the penultimate layer (‘AvgPool’) of the ResNet-50 architecture as the final outputs (2048 features). These features were then used to train and test SVM classifiers.

#### Training SVM classification

For ResNet-50, feature outputs from the penultimate layer were fed into the SVM classifier with a linear kernel. We then appended the image labels and correct binary outputs to the output features, generating complete training datasets. We applied a leave-one-out cross validation to split training data with test data (k-fold = 5). For the image sets, we trained on all objects (10 total) while withholding one test object. To prevent potential overfitting and to increase the robustness of the SVM model, the training dataset was resampled 1000 times to ensure a more robust and varied training dataset. A regularization parameter (C) was included to achieve a balance between overfitting and underfitting. Output features were dimension reduced by PCA such that the output features explained by 90% of variance were retained as final outputs. For geometric models, we applied the same procedure except that the features were geometric features (height, base, T/B ratio, and centroid) in the feature dataset. As with ResNet-50, correct binary outputs for each stimulus were trained. The training iterated all objects until every object served as a test object.

#### Testing SVM classification

A binary prediction (1 = fall onto the green mat; 0 = fall onto the red table) on each test object at each tilt was obtained from SVM classification. The binary predictions were then aggregated across all resampling data to generate a full dataset with predicted values on all objects and angles. We examined both the confusion matrices and ROC curves of each model to ensure the quality of training.

### Supplementary Information

Supplementary Information.

## Data Availability

All stimuli and data are available at: https://osf.io/q4yce/.
