# Supplementary material for: Object geometry serves humans’ intuitive physics of stability
Source: Sci Rep. 2024 Jan 19;14:1701. doi: 10.1038/s41598-024-51677-5 (PMC10799025; doi:10.1038/s41598-024-51677-5)
Supplement: Supplementary file 1 — Supplementary Information. [file 41598_2024_51677_MOESM1_ESM.docx]

**Supplementary Information**

***Table 1.*** Formulas for calculating the centroid of each object and the resulting ground truth critical angle. See OSF (<https://osf.io/q4yce/>) for supplemental scripts.

| Object | Centroid height | Critical Angle |
| --- | --- | --- |
| **Conical frustum**  **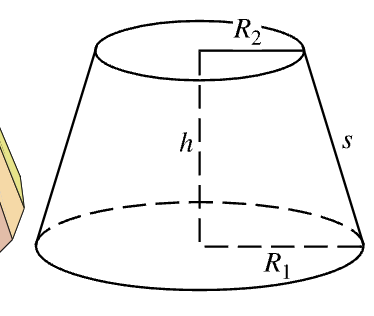** | *h_centroid_* = $\frac{{h(R}_{1}^{2}+2R_{1}R_{2}+3R_{2}^{2})}{4\left( R_{1}^{2}+R_{1}R_{2}+R_{2}^{2} \right)}$  R_1_: Larger radius  R_2_: Smaller radius | $\theta_{critical}$ *= ArcTan* $\frac{h_{centroid}}{r}$ |
| **Cone**  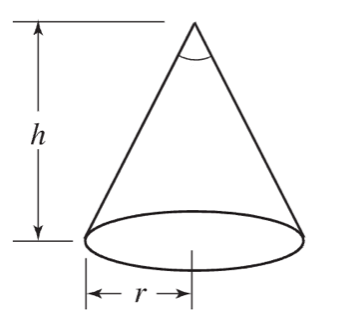 | *h_centroid_* = $\frac{1}{4}h$ |  |
| **Cylinder and cuboid**  *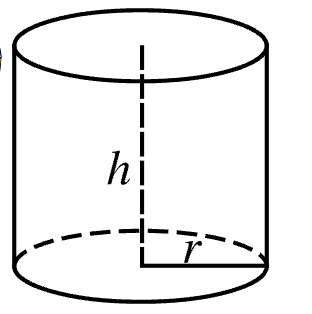* | *h_centroid_* = $\frac{1}{2}h$ |  |

**Age group analyses**. In Experiment 1A, to test for potential differences between child and adult participants in PSE estimates, an ANOVA (Greenhause-Geisser corrected) was performed with object (within-subject) and age group (between-subject) as factors. This analysis revealed a significant main effect of object, *F*(2.64, 72.04) = 140.60, *p* = .792, *η_p_^2^* = .792, and a significant interaction between object and age group (*p* < .001, *η_p_^2^* = .245), such that children had lower PSE estimates compared to adults for the object with the lowest aspect ratio (*M_children – adults_* = -11.99°, *t*[37] = - 2.85, *p* = .007, *d* = .94). No other age differences in PSE estimates were found for other objects.

Experiment 1B similarly revealed a significant main effect of object, *F*(1.3, 41.68) = 72.68, *p* < .001, *η_p_^2^* = .694. There was no effect of age group (*p* = .686, $\eta_{p}^{2}$ = .005), nor an interaction between object and age group (*p* = .059, *η_p_^2^* = .098).

Experiment 2A revealed no significant effect of object, *F*(1, 36) = .023, *p* = .367, $\eta_{p}^{2}$ = .023, BF_10_ = .36, age group (*p* = .088, *η_p_^2^* = .079, BF_10_ = 1.2), nor an interaction between object and age group (*p* = .556, $\eta_{p}^{2}$ = .010, BF_10_ = 0.38). The bayes factor for age group suggests moderate evidence of age differences in PSE estimates, such that adults’ PSE estimates were higher than those of children’s (*M*_difference_ = 3.27°).

In Experiment 2B, there was a significant effect of object , *F*(1, 39) = 4.58, *p* = .039, *η_p_^2^* = .105, and a significant object by age group interaction, *F*(1, 39) = 15.65, *p* < .001, $\eta_{p}^{2}$ = .039. Post hoc analyses revealed that children’s PSE estimates were significantly lower than those of adults’ for both objects (the left object in Supplemental Figure 3-2B: *M_children – adults_* = -4.79°, *t*[39] = -2.99, *p_corrected_* < .001, *d* = .96; the right object in Supplemental Figure 3-2B: *M_children – adults_* = -9.72°, *t*[39] = -4.66, *p_corrected_* < .001, *d* = 1.49).

*Child sample.* One-way ANOVAs were performed to examine the PSE estimates of 5- and 6-year-olds in Experiment 1A. The results revealed no significant differences in the two age groups in Experiment 1A, *F*(1, 68.7) = .31, *p* = .581; Experiment 1B, *F*(1, 9.75) = 2.94, *p* = .12; Experiment 2A, *F*(1, 35.8) < 1, *p* = .683, and Experiment 2B, *F*(1, 43.5) = .02, *p* = .887. Thus, our analyses combined these two age groups in the child sample.

*Order effect.* Because a within-subject design was used for Experiments 2A and 2B among adults, we tested for an effect of order. The results revealed no order effect, *F*(1, 16) = .034, *p* = .855, and thus, this variable was not considered further.

***Supplemental Figure 1.*** Accuracy of adult and child participants in Experiments 1 and 2. In both experiments, adults performed better than children (*ps* < .001; Exp. 1A: $\eta_{p}^{2}$ = .37; Exp. 1B: $\eta_{p}^{2}$ = .45; Exp. 2A: $\eta^{2}$= . 27; Exp. 2B: $\eta^{2}$= . 54). Error bars represent 95% CIs.

***Supplemental Figure* 2.** Differences in PSEs for human participants and models compared to the ground truth values of critical angles by experiment (top) and object (bottom). Underestimation corresponds to a prediction that an object falls at a smaller angle than the critical angle. Overestimation corresponds to a prediction that an object falls at a larger angle than the critical angle.

**
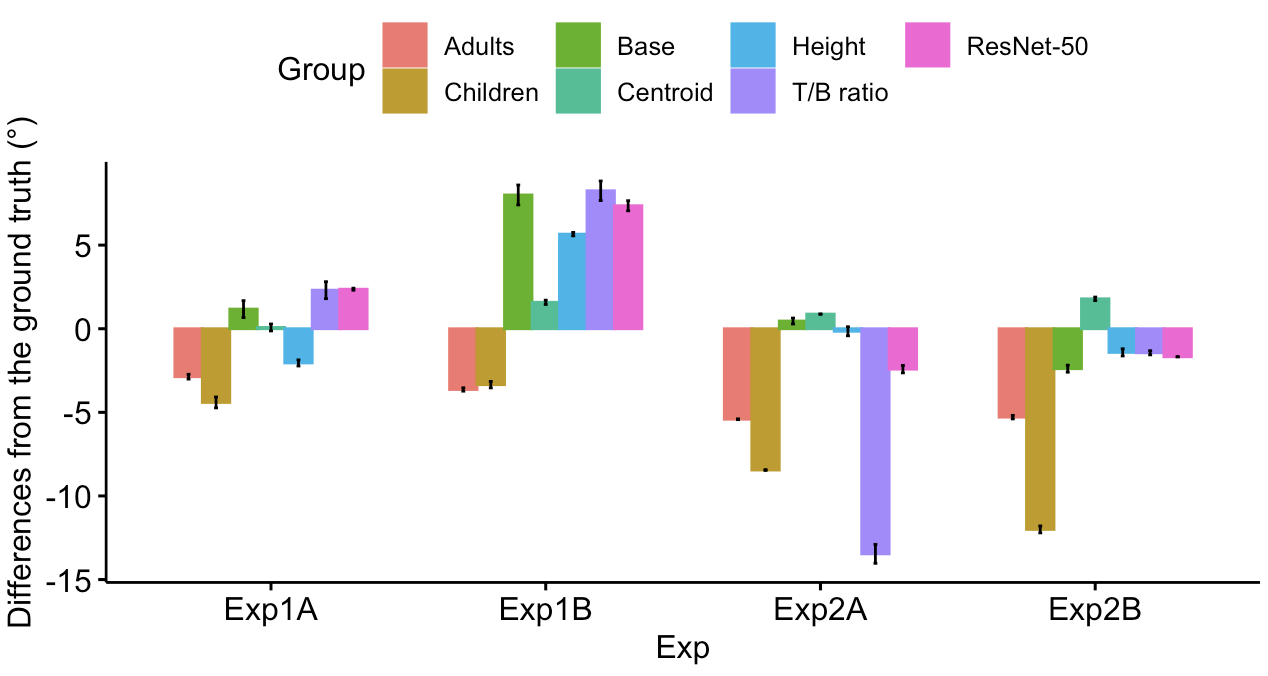
**

**
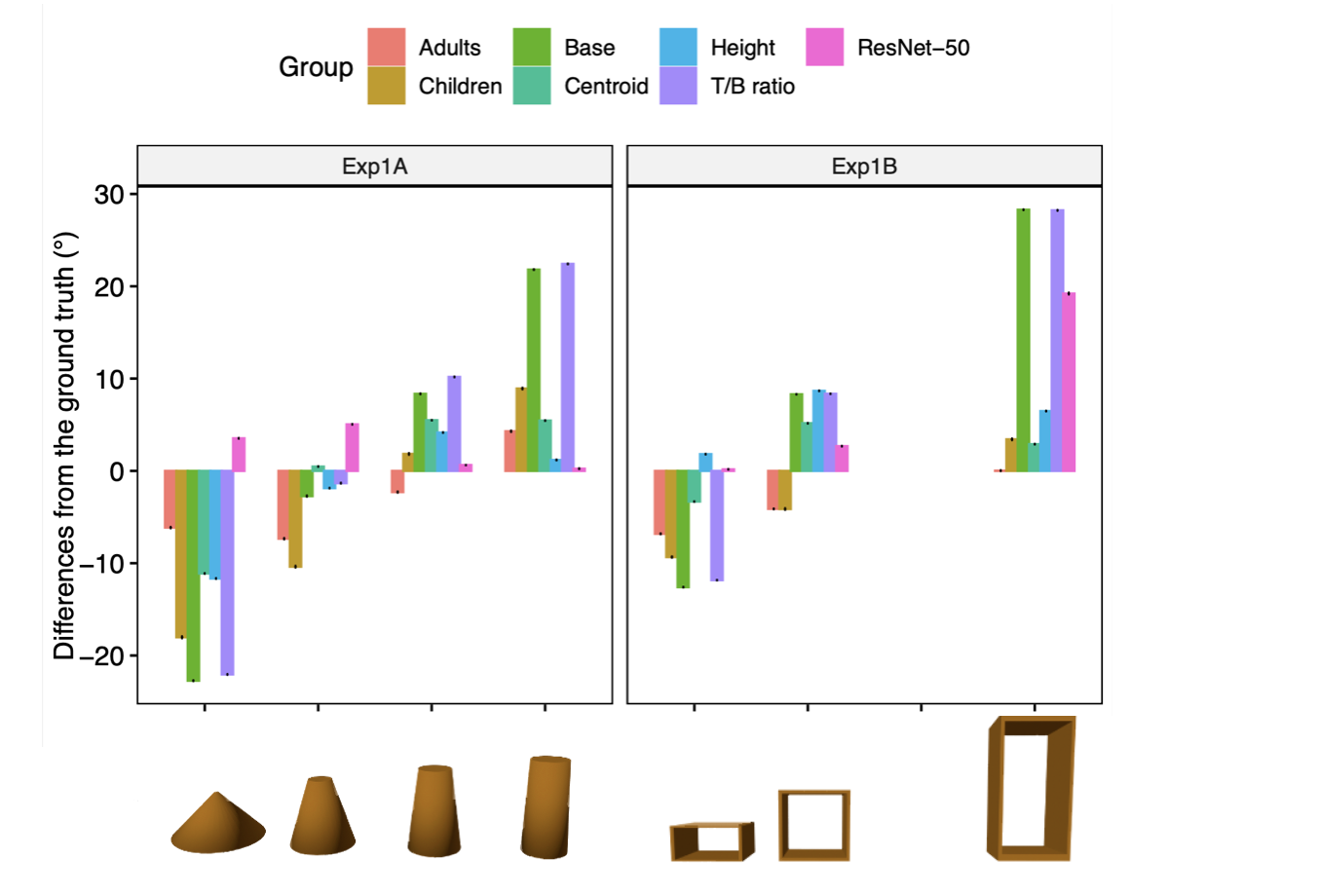
**


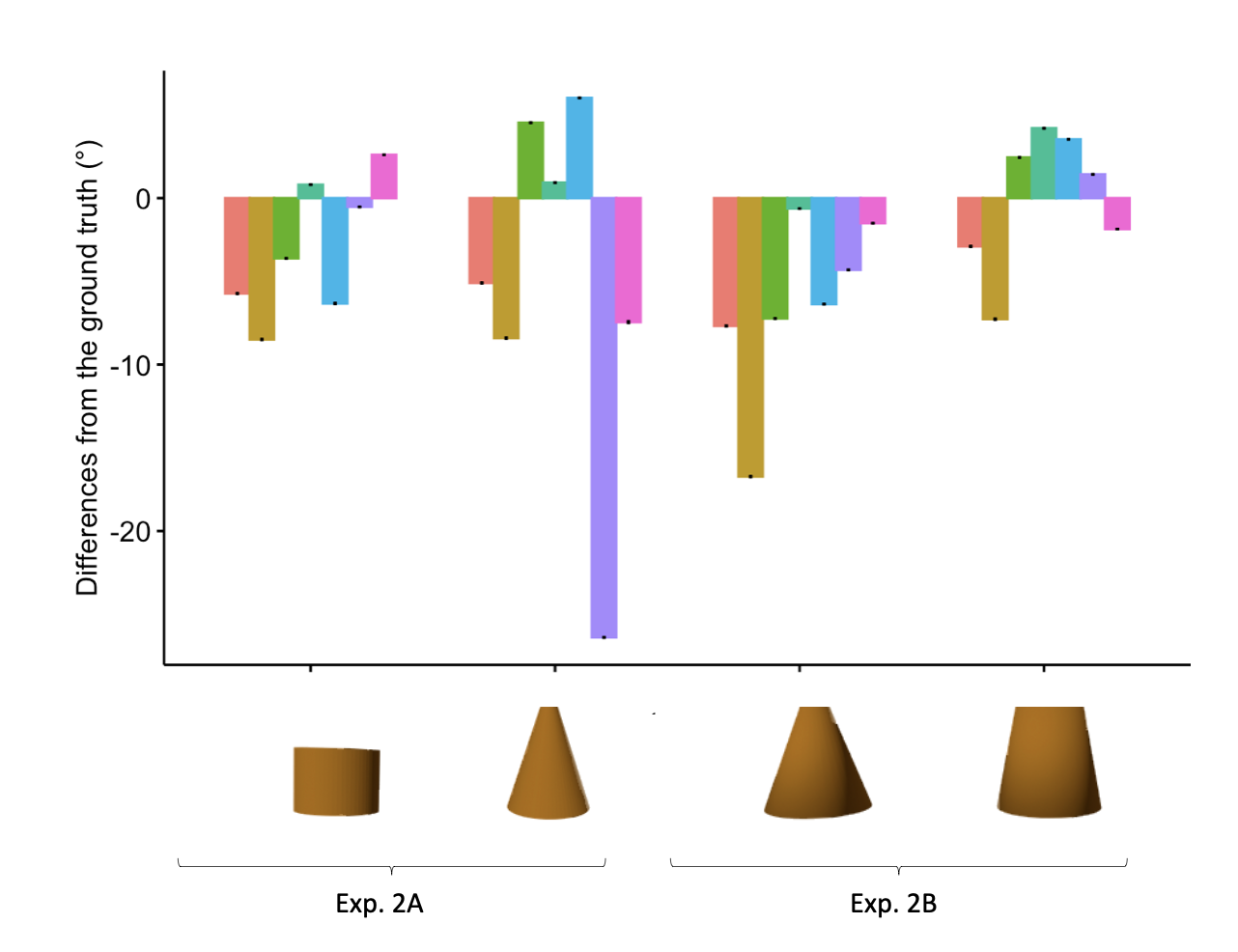


***Supplemental Figure* 3.** (A) Psychometric functions for human participants by experiment and age group. See OSF (<https://osf.io/q4yce/>) for GLM fitting from individual participants. (B) Psychometric functions for each model in each experiment.


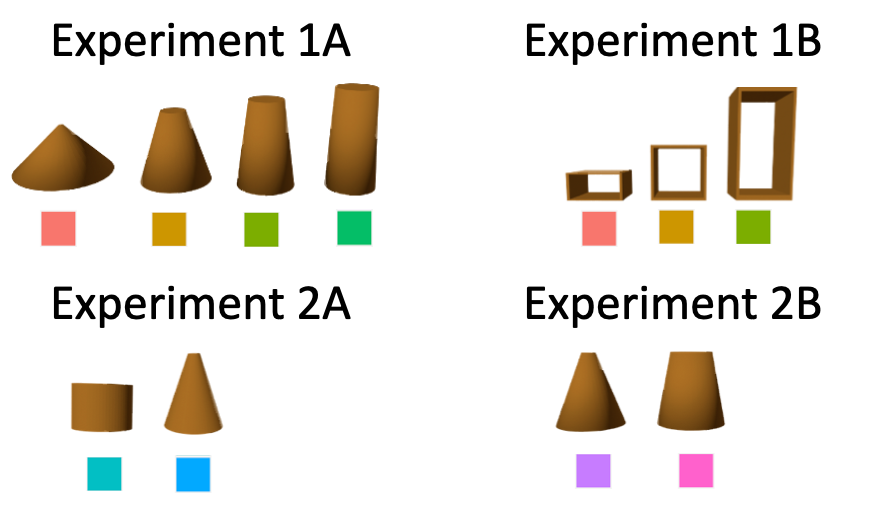


**A**

**
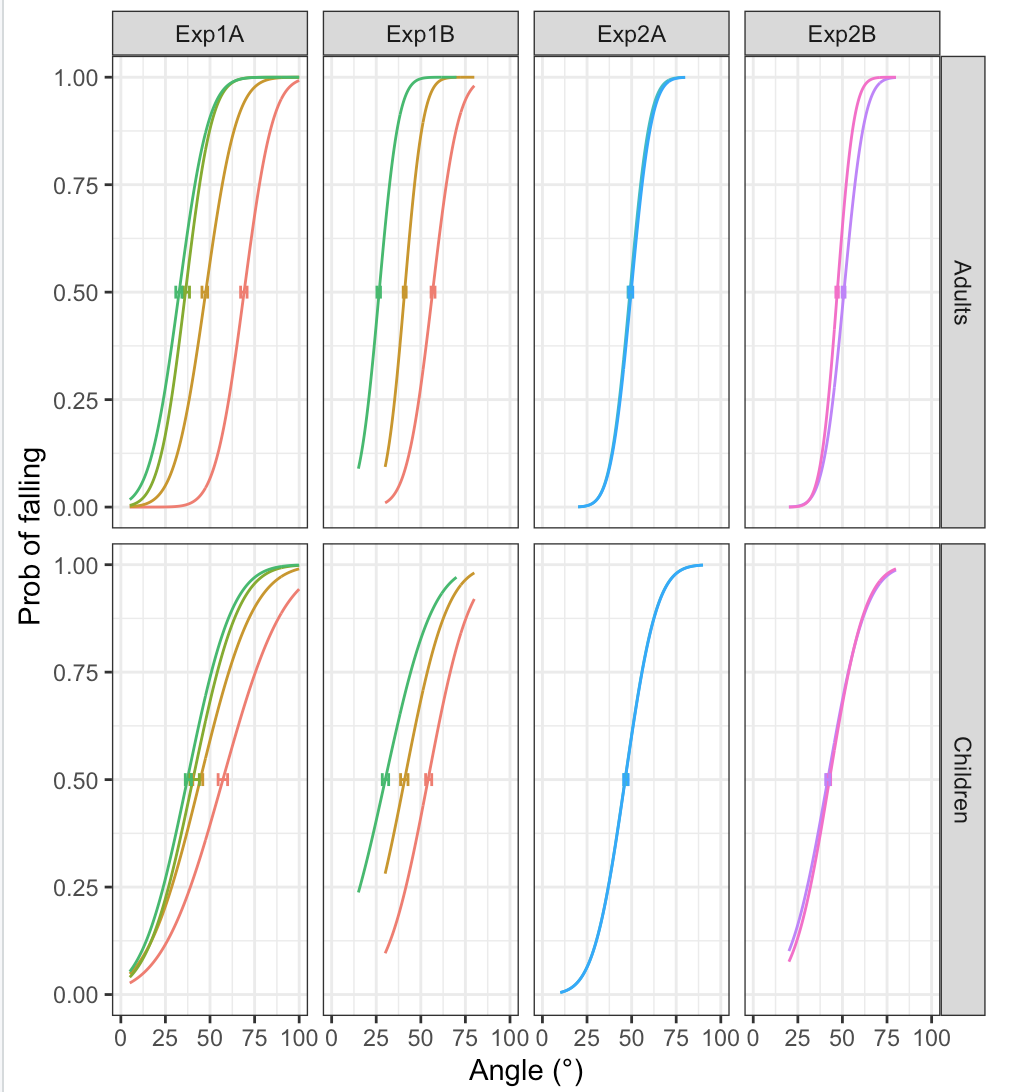
**

**B**


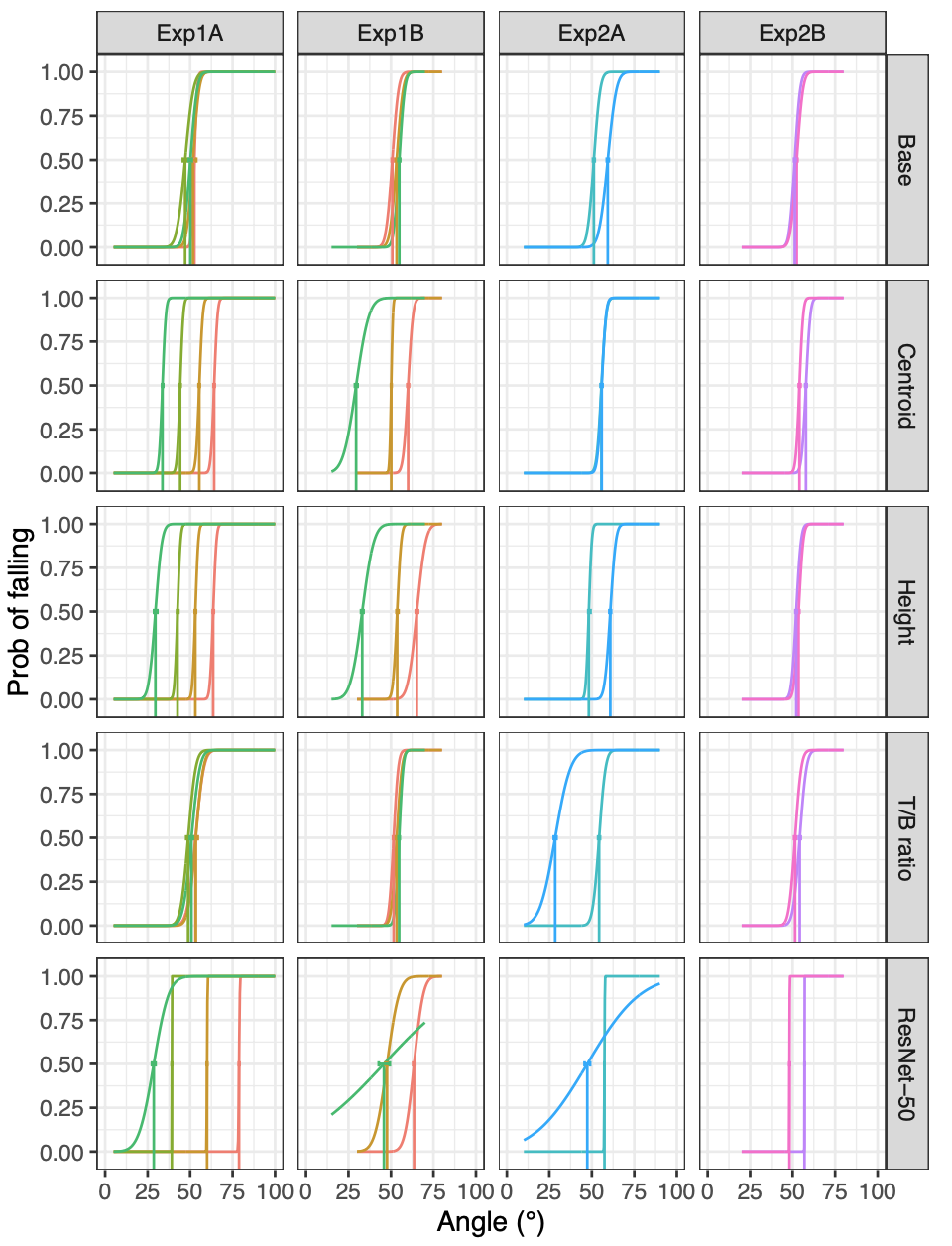


**Human observers’ reaction times.** Although the findings in the main text suggest that human performance is well modeled by object geometry, it is still plausible that mental simulation, consistent with an IPE, plays a role in judging the stability of a tilted object. To address this possibility, we compared human participants’ reaction times (RTs) when deciding between falling to the ground or onto the table. From the perspective of an IPE, falling to the ground should take longer than falling onto the table because the distance in the former is longer than in the latter.

RTs were preprocessed, with those exceeding 10 s and 2.5 SDs trimmed at the participant level. Because RTs tend to vary as a function of the tilted angle, angle was entered as a covariate. An analysis of covariance (ANCOVA), with response type and age group entered as fixed factors, revealed a significant effect of angle (*F*[1, 2677] = 6.04, *p* = .014) and age group (*F*[1, 2677] = 480.35, *p* < .001). Importantly, however, there was no significant difference between falling to the ground or onto the table (*F*[1, 2677] = 1.47, *p* = .224, BF_01_ = 10.87; Supplemental Figure 4A). Falling to the ground did not take longer (M = 2.24 s) than falling onto the table (M = 2.29 s), which suggests that mental simulation did not play a significant role in participants’ responses on this task. To ensure that the observed effects were not due to noise from incorrect trials, we further analyzed only the correct trials in which only human judgments aligned with the ground truth judgments (resulting in 15% trial loss). The results revealed a significant difference between falling to the ground versus onto the table (*F*[1, 2071] = 15.19, *p* < .001), but in the opposite direction of what would be predicted by a mental simulation account. Specifically, RTs were shorter for the longer distances (i.e., falling to the ground; M = 2.14 s) than the shorter distances (i.e., falling onto the table; M = 2.34 s).

Nevertheless, one concern with these findings is that uncertainty at the critical angles may have resulted in longer RTs, potentially obscuring a simulation effect. To address this concern, the proportion of responses of falling to the ground at 50% were trimmed from the analyses. We then compared lower proportion of falling in this case (0% <= proportion < 50%) with higher proportion of falling (50% < proportion <= 100%). Results revealed no significant difference between these possibilities (*F*[1, 2237] = .27, *p* = .602, BF_01_ = 14.28; Supplemental Figure 4B), consistent with the previous analyses and contra a simulation account. Additionally, when the correct trials only were analyzed, RTs for the lower proportion of falling were significantly longer (2.30 s) than those for the higher proportion of falling (2.11 s; *F*[1, 2303] =14.98, *p* < .001), which is inconsistent with mental simulation for falling direction.

***Supplemental Figure 4.*** RT data analyzed as a function of the type of support base (A) and the type of proportion of falling responses (B).

**
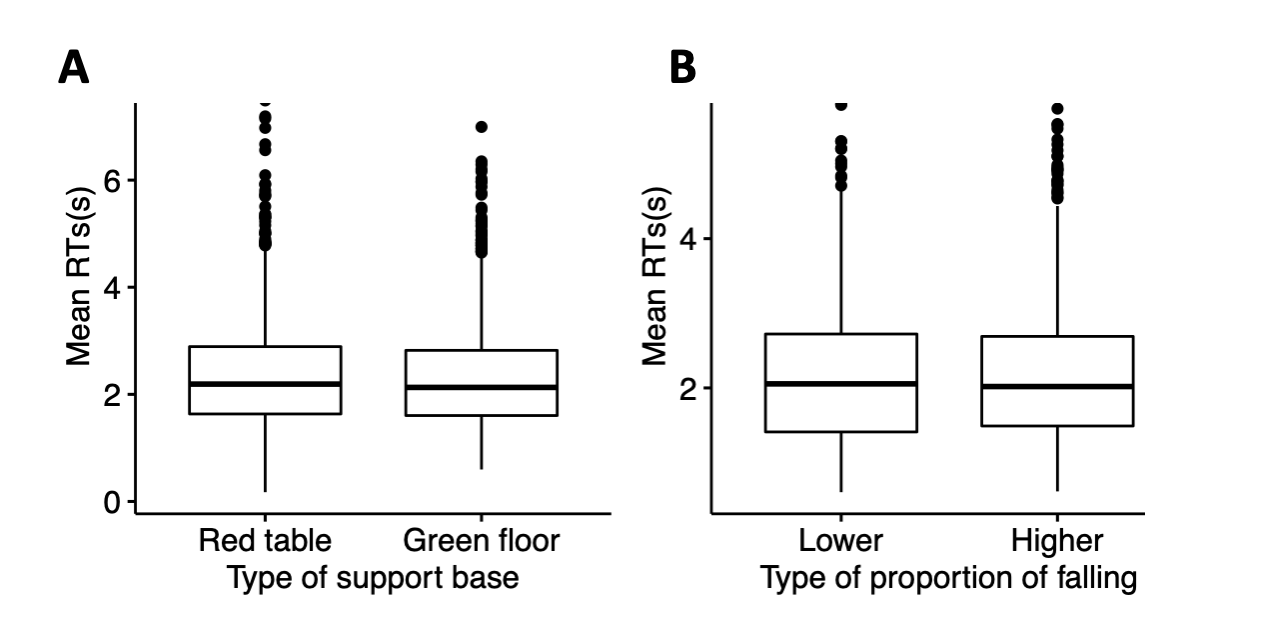
**

***Supplemental Figure* *5*.** RT data analyzed as a function of proportion of falling responses. (A) Proportion of falling restricted to lower probabilities (< 50%). (B) Proportion of falling restricted to higher probabilities (> 50%).


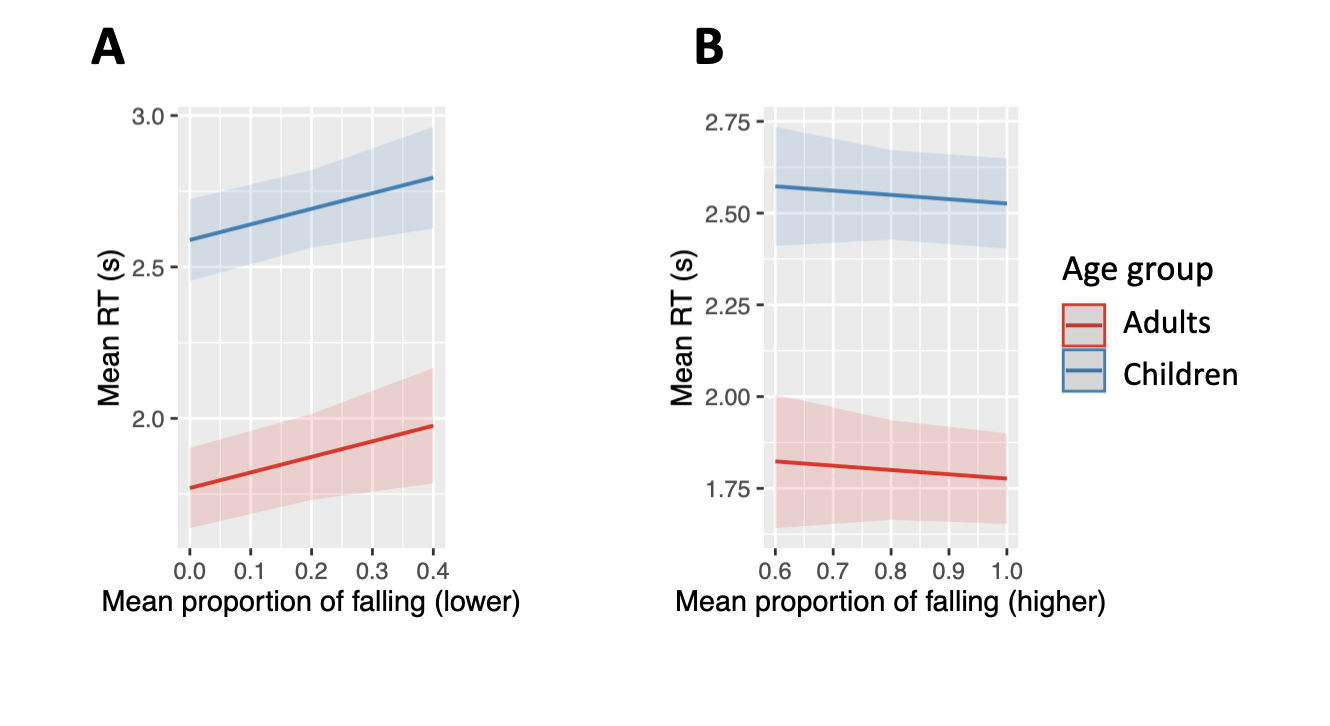


Even though we did not find any group differences between the two potential types of simulation (stay or fall), it remains likely that RTs would scale as a function of proportion of falling responses, given the uncertainty associated with decisions that made on tilted angles away from the extremes. Separate linear mixed analyses were performed on the two types of proportion of falling data (low versus high), with the proportion of falling, age group, and angle entered as fixed factors and participant as random factor. Proportion of falling at 50% was excluded.

Results revealed that proportion of falling responses was a significant predictor of RT in the low proportion case (*b* = .51, *p* = .014; see Supplemental Figure 5A). This was not the case, however, in the high proportion case (*b* = -.12, *p* = .545; see Supplemental Figure 5B), suggesting RTs did not scale as a function of certainty (farther from the 50% threshold). Assuming that high certainty would involve fewer numbers of simulations, and consequently shorter RTs, the current findings do not appear to support this prediction, at least in trials having a higher proportion of falling. However, an important caveat is that in both types of falling responses, angle was a significant predictor (*ps* < .001), suggesting RTs scaled as a function of distance.

**Acknowledgements**

We thank So Ye Han and Anna Bulka for their assistance with data collection.

**Author contributions statement**

Y.L.: Conceptualization, methodology, data curation, formal analysis, writing - original draft. V.A.: methodology, writing - review and editing, resources. S.F.L.: Conceptualization, methodology, writing - review and editing, resources, supervision. All authors approved the submission.

**Additional information**

**Supplemental information:** Supplemental information is available.

**Competing interests:** The authors declare no competing interests.
